# Restoration of a Stuck Leaflet

**DOI:** 10.1002/ccd.31550

**Published:** 2025-05-08

**Authors:** Elisa Tomarelli, Gianluca Di Pietro, Gennaro Sardella, Massimo Mancone

**Affiliations:** ^1^ Department of Clinical, Internal, Anesthesiological and Cardiovascular Sciences, Umberto I Hospital Sapienza University of Rome Rome Italy

**Keywords:** aortic regurgitation, aortic stenosis, transcatheter aortic valve implantation, post‐TAVI complications, stuck leaflet

## Abstract

Severe aortic regurgitation (AR) for a leaflet malfunction after transcatheter aortic valve implantation (TAVI) is a dreadful complication that can lead even to cardiogenic shock. New valve's design has reduced the rate of paravalvular regurgitation, however central regurgitation is still a challenging complication. An 88‐year‐old woman with severe aortic stenosis underwent TAVI with an Edwards Sapien III valve. After the procedure, angiography revealed third‐degree angiographic central AR, causing hemodynamic instability. The team suspected a stuck leaflet and corrected it with catheter manipulation. The patient's condition stabilized, and follow‐up echocardiography showed proper valve position and function. Severe AR following TAVI, while rare, can result in critical clinical deterioration. Recent evidence suggest that even mild grade of AR post‐TAVI can affect long‐term outcomes. This case has the aim to promote a prompt diagnosis and intervention on a stuck leaflet to reduce patient morbidity, hospitalization time, and resource utilization. This case illustrates the warning scenario of a severe central AR for a stuck leaflet after TAVI. Prompt recognition and management of this complication are crucial to improving clinical outcomes.

## 1 Introduction

Transcatheter aortic valve implantation (TAVI) has revolutioned the treatment of aortic valve stenosis in all class of surgical risk [1, 2, 3]. Aortic regurgitation (AR) following TAVI is recognized as a challenging complication that can significantly impact patient's outcomes increasing mortality and morbidity, even in a mild grade [4].

AR post‐TAVI could occur due to several factors including improper valve positioning, underexpansion of the prosthesis, or anatomical characteristics such as a bicuspid aortic valve or significant calcification [5].

This case report aims to detail the presentation, diagnosis, and management of a patient who presented severe AR immediately after TAVI. Sharing this case we hope to provide insights into the potential mechanisms of AR after TAVI and discuss strategies to overcome this complication.

## 2 Case Report

**Figure 1:**
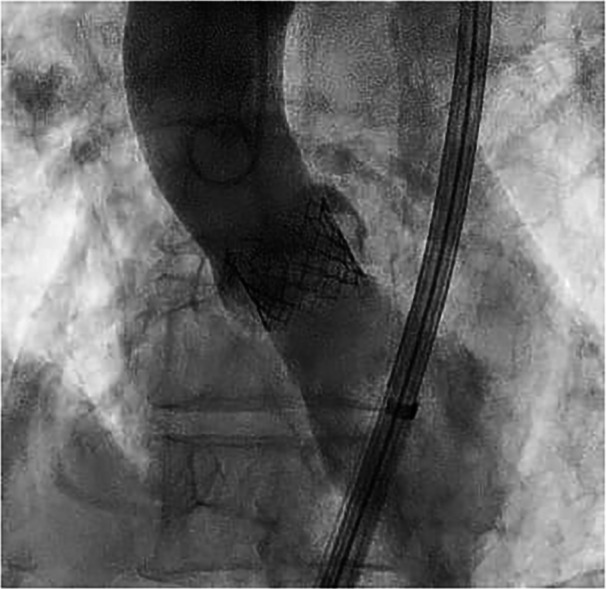
Severe aortic regurgitation after bioprosthesis implantation.

**Figure 2:**
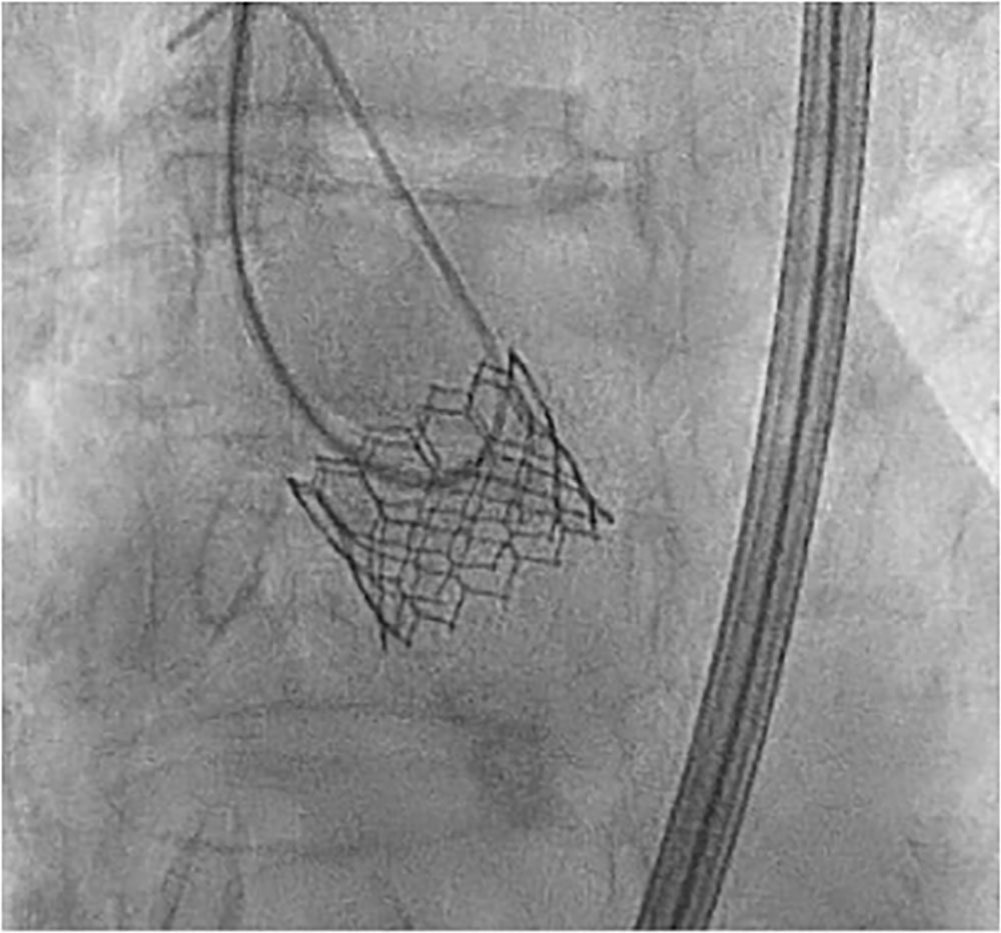
Solicitation of the stuck leaflet.

**Figure 3:**
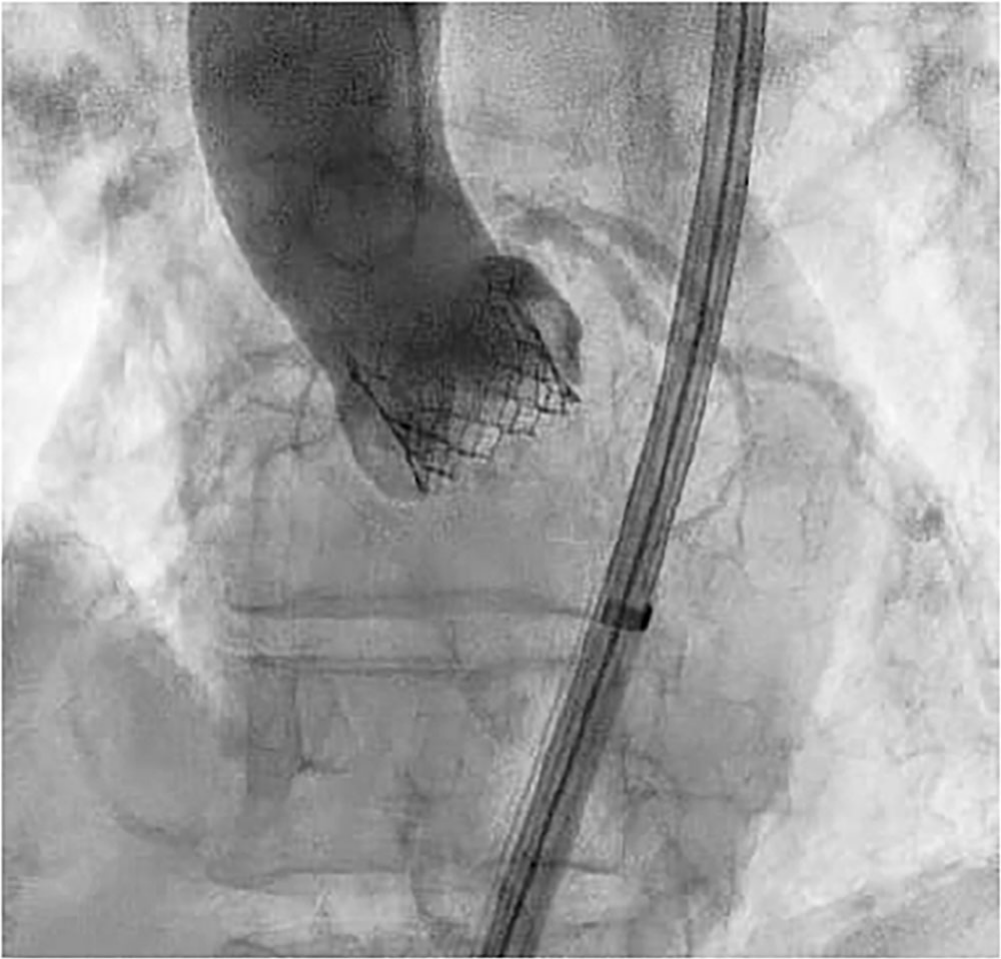
Absence of regurgitation after the restoration of the leaflet motion.

An 88‐year‐old female patient was admitted for exertional dyspnea (New York Heart Association class III). The transthoracic and transoesophageal echocardiography showed a preserved biventricular systolic function and a severe paradoxical aortic stenosis normal flow/low gradient with planimetric aortic valve area (AVA) 0.6 cm^2^, functional AVA 0.6, aortic valve peak velocity (V_max_) = 3.9 m/s, aortic valve peak pressure gradient (PG) = 60 mmHg, aortic valve average pressure gradient (MG) 38 mmHg, stroke volume (SV) index 58 mL/m^2^. After Heart Team evaluation (Euroscore II 7%), the patient was scheduled for TAVI. An intraprocedural coronary angiography showed a nonsignificant coronary artery disease. The procedure was performed through a transfemoral approach, under sedation. An aortic angiography was performed with a pig‐tail advanced through right radial access (6 French) and showed a calcific valve with reduced systolic leaflet movement. A Safari guidewire was advanced into the left ventricle through the right femoral artery and the Edwards Commander delivery System was introduced through the Edwards eShealth Introducer Set (14 French). The bioprosthesis release (20 mm Edwards Sapien III) was performed under rapid on‐the‐wire pacing (180 beats per min with significant systolic pressure drop). The angiographic control showed a correct positioning of the valve with a third‐grade angiographic central regurgitation (Figure 1, Video S1). This led to the worsening of the patient's hemodynamic conditions with hypotension, respiratory failure, and hyperlactacidemia. The correct positioning of the prosthesis along with the absence of perivalvular leaks, led us to conclude that the malfunction was likely intraprosthetic. During the procedure, it was not possible to clearly identify the specific leaflet involved in the AR due to the unavailability of transesophageal echocardiography at the time of the complication and the poor image resolution of transthoracic echocardiography. However, angiographic evaluations suggested that the left‐sided leaflet was less mobile compared to the right‐sided one, and the regurgitant jet appeared to originate from that region. Therefore, we decided to pursue a direct approach to what we believed was the underlying cause of the regurgitation, before resorting to a rescue procedure, such as the valve‐in‐valve technique previously described in the literature. So after re‐advancing the pigtail over a standard 35 angiographic guidewire at the valvular plane, with a delicate and repetitive solicitation of the stuck leaflet, the normal excursion was restored (Figure 2, Video S2). The final angiographic and transthoracic echocardiographic control showed correct positioning of the valve, with no peri‐ or trans‐valvular leak (Figure 3, Video S3). Subsequently, a progressive improvement in clinical and hemodynamic conditions was observed. The echocardiographic control showed correct positioning of the valve without significant gradient (V_max_ 1.92 m/s, MG 10 mmHg) and peri‐ or trans‐valvular leak. The patient's hospitalization continued without complications until discharge 2 days after the procedure.

## 3 Discussion

The case described above is a rare occurrence, but it can lead to a severe deterioration of the patient's clinical and hemodynamic condition, as already described in the literature in a similar situation that was resolved with a valve‐in‐valve intervention [6, 7]. TAVI has the greatest rate of AR than surgical aortic valve [2, 8, 9]. Further studies are needed to understand the actual long‐term outcomes of AR. However, recent data from a 5‐year study of patients who underwent TAVI have shown that even mild AR can have an impact on clinical outcomes [10]. Even though the intrinsic properties of the Edwards Sapien III, with its new outer polyethylene terephthalate sealing cuff, significantly reduce paravalvular regurgitation, they are not able to prevent occurrences like the one described above [11]. Therefore, by sharing this case, we hope to raise pratictioners' awareness about the possibility of this complication aiming for its intraprocedural prompt diagnosis and treatment, to reduce morbidity, hospitalization time, and resource expenditure.

## Conflicts of Interest

The authors declare no conflicts of interest.

## Supporting information

Video 1: Severe aortic regurgitation after bioprosthesis implantation.

Video 2: Solicitation of the stuck leaflet.

Video 3: Absence of regurgitation after the restoration of the leaflet motion.

## Data Availability

The data that support the findings of this study are available from the corresponding author upon reasonable request.
